# Integration efficiency for model reduction in micro-mechanical analyses

**DOI:** 10.1007/s00466-017-1490-4

**Published:** 2017-11-10

**Authors:** Rody A. van Tuijl, Joris J. C. Remmers, Marc G. D. Geers

**Affiliations:** Eindhoven University of Technology, P.O. Box 513, Eindhoven, The Netherlands

**Keywords:** Model reduction, Micro-mechanics, Empirical interpolation method, Empirical cubature method, Homogenization

## Abstract

Micro-structural analyses are an important tool to understand material behavior on a macroscopic scale. The analysis of a microstructure is usually computationally very demanding and there are several reduced order modeling techniques available in literature to limit the computational costs of repetitive analyses of a single representative volume element. These techniques to speed up the integration at the micro-scale can be roughly divided into two classes; methods interpolating the integrand and cubature methods. The empirical interpolation method (high-performance reduced order modeling) and the empirical cubature method are assessed in terms of their accuracy in approximating the full-order result. A micro-structural volume element is therefore considered, subjected to four load-cases, including cyclic and path-dependent loading. The differences in approximating the micro- and macroscopic quantities of interest are highlighted, e.g. micro-fluctuations and stresses. Algorithmic speed-ups for both methods with respect to the full-order micro-structural model are quantified. The pros and cons of both classes are thereby clearly identified.

## Introduction

In recent years the use of composite or multi-phase, high performance steels and hybrid materials in advanced structures has increased significantly. Due to the ability to tailor their mechanical properties, these materials are applied in high-tech systems and structures where optimal material properties such as a high strength to weight ratio are essential. To achieve these excellent properties, the underlying microstructures in these heterogeneous materials tend to become increasingly complex, with strongly non-linear behavior of the constituents.

As a consequence, the material testing procedures have become intricate, time-consuming and expensive. The number of required experiments can be reduced drastically by reliable modeling of the material behavior. Computational homogenization is one of the most accurate modeling techniques currently available to analyze and design the material behavior of heterogeneous materials on a structural scale. Microstructures of heterogeneous materials can be modeled adequately, but it remains a computationally demanding problem when applied on materials with a large scale separation between the structural dimensions and the dimensions of the material heterogeneities.

The early concepts of computational homogenization were introduced by Suquet [1] and extended and refined by several authors over the years, e.g. Renard and Marmonier [2], Guedes and Kikuchi [3] and Terada and Kikuchi [4]. The framework captures the behavior on the macroscopic scale by solving a boundary value problem on a representative volume element of the microstructure of the material. Smit et al. [5] adapted the framework to account for large deformations and rotations. Miehe et al. [6] combined this framework with a crystal plasticity material model to study texture development in polycrystalline metals. By means of a volume averaging technique, the homogenized relations between stress, stiffness and strain can be found [7]. Feyel and Chaboche modeled a SiC/Ti composite material using computational homogenization in [8], where the term FE${}^2$ was coined for the first time. A second-order computational homogenization framework which inherently accounts for the size of the microscale model was proposed by Kouznetsova et al. [9]. More recently, computational homogenization has been used in various fields to analyze the material behavior, such as acoustics [10], composites [11] among many other fields. A detailed overview of the advances in computational homogenization is presented by Geers et al. [12] and Matouš et al. [13].

The common denominator in the aforementioned models is that the material behavior on the macroscale is obtained from a detailed model of the microstructure of the material. When the microscopic model is sufficiently large and detailed to accurately capture the homogenized behavior of the material it is referred to as a representative volume element (RVE). In solving a macroscopic problem one can use the computationally homogenized model of the representative volume element or in materials design analysis one can use the RVE to analyze the resulting macroscopic response of different microstructures. This requires the same microscopic RVE problem to be resolved numerous times for different load-cases.

One approach to decrease the required computation time is to increase the efficiency of solving the microscopic system of equations, Michel et al. [14] used a Fast-Fourier Transform to reduce the computational costs of solving a stress-controlled microscopic problem. Another approach is to reduce the number of unknowns of the microscopic model. This can be achieved by using a more suitable global basis to solve the problem as done using a classical Rayleigh-Ritz technique [15].

This reduced order modeling technique was originally proposed in the late 70’s begin 80’s with Almroth et al. and Noor et al. [16, 17]. The global basis is generated a-priori from a local basis using Proper Orthogonal Decomposition (POD) [18] going back to the works of Pearson [19] and Schmidt [20]. Variants to this technique are known as principle component analysis (PCA) [21], Karhunen–Loève transform (KLT) [22, 23], proper orthogonal eigenfunctions [24], factor analysis [25] and total least squares [26]. The procedure of finding the global basis is strongly related to the singular value decomposition [26].

The number of global basis functions required to capture the solution accurately is typically orders of magnitude smaller than the number of local basis functions used in classical discretization techniques such as the Finite Element Method. Rewriting the problem onto the global basis reduces the size of the algebraic system of equations that needs to be resolved at every iteration. A detailed overview of the advances in reduced order modeling is presented in [27] and [28].

As pointed out by Rathinam and Petzold [29] the reduction of the number of degrees of freedom does not necessarily result in a reduction of the computational costs as the integration of the nonlinear terms in the model is not tackled by the reduced basis approach. To resolve the high computational costs of evaluating the nonlinear integrand, a number of methods have been proposed, such as Proper Generalized Decomposition [30], which reduces the costs through separation of variables. Transformation Field Analysis is another semi-analytical approach, introduced by Dvorak [31], in which the computational costs of the FE${}^2$ scheme are reduced by assuming constant plastic strain fields. The method was later extended by Michel and Suquet [32] to account for non-uniform plastic strain fields. As pointed out by Hernández et al. [33], hyper-reduction techniques can be divided into two categories; (1) models reducing the costs by interpolation of the nonlinear terms, i.e. *Empirical Interpolation Methods* (EIM) and (2) models reducing the costs of the integration scheme, minimizing the number of evaluations of the non-linear term required, i.e. *Cubature Methods*.

**Fig. 1:**
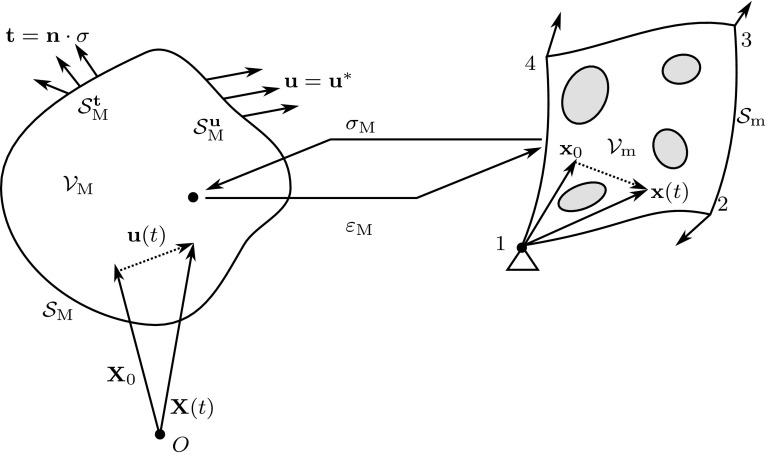
Outline of the macro- and microscopic problem

*Empirical interpolation* of the nonlinear stress is done by projecting locally sampled values, also known as *gappy data*, onto a basis for the nonlinear term. The reconstruction of gappy data using modes is pioneered by Everson and Sirovich [34] from which several techniques originated such as the Empirical Interpolation Method [35], and later the Best Point Interpolation Method (BPIM) [36], Missing Point Estimation (MPE) [37], Discrete Empirical Interpolation Method (DEIM) [38]. A comparison between different interpolating methods is given by Soldner et al. [39]. This review considers a geometrically non-linear hyper-elastic RVE which is reduced using three hyper-reduction methods, namely Discrete Empirical Interpolation Method (DEIM), Gappy-POD and Gauss-Newton with Approximated Tensors (GNAT). The focus of the review by Soldner et al. lies on the robustness of the methods.

On the other hand, *cubature methods* reduce the cost of the integration of the nonlinear integrand by using a reduced set of integration points (or elements) with weights that minimize the integration error. The first cubature method was proposed by An et al. [40] and later Farhat et al. [41] introduced the *Energy-Conserving Sampling and Weighting (ECSW) hyper reduction method* in the field of computational mechanics.

This paper studies the accuracy and efficiency of hyper-reduction techniques in the context of microstructural volume element analysis. To this purpose, a detailed comparison will be made between the Empirical Interpolation Method on the one hand and the Cubature Method on the other hand. We will compare two specific implementations of these methods, namely the High-Performance Reduced Order Modeling technique (HP-ROM) [42] and the Empirical Cubature Method [33], respectively. Both implementations are selected for their capability to handle the singularities arising when hyper-reduction techniques are applied to microstructural models loaded through macroscopic prescribed fields.

In this comparison emphasis is given on the reduction of the computational costs of evaluating the nonlinear stress, the efficiency in terms of computational time and accuracy in resolving the RVE problem. In particular, this comparison will focus on the path-dependency originating from the elasto-viscoplastic material behavior.

The paper is organized as follows. In Sect. 2, a small-strain computational homogenization procedure is outlined, after which the standard Reduced Order Modeling approach is introduced in Sect. 3. In Sect. 4 the two classes of hyper-reduction will be explained briefly. The core of this paper is the evaluation of different hyper-reduction methods to resolve the expensive computation of the stress field, as presented in Sect. 5. The conclusions will be presented in Sect. 6.

## Computational homogenization

In this section, the two-scale computational homogenization procedure will be outlined briefly. In the computational homogenization framework a high-fidelity problem at the macroscopic length scale $l_\mathrm{M}$ involving microscopic details with a characteristic length scale $l_\mathrm{m}\ll l_\mathrm{M}$ is decomposed into two boundary value problems at separate scales, as schematically depicted in Fig. 1. By decomposing the problem into a micro- and macroscale problem, the numerical costs of solving the full-scale problem are reduced drastically.

At the macroscale, where the variables and fields are denoted by subscript $\mathrm{M}$, the constitutive behavior is derived from the homogenized response of a Representative Volume Element living at the microscale. Computational homogenization is easily formulated as a deformation driven problem, i.e. the RVE is loaded with a deformation tensor from a macroscopic point, where the resulting stress tensor is required. Using a Hill–Mandel based homogenization procedure, the macroscopic stress can be derived from the microscopic stress-field of the RVE. The macroscale problem, the microscale problem and the coupling will be outlined subsequently.

### Macroscale problem

The macroscopic problem is defined as a material body $\mathcal {V}_\mathrm{M}$ which consist of a heterogeneous material with a particular microstructure. A material point at the macroscopic scale is described by its position vector ${\mathbf {X}}(t)$. The position vector on the reference configuration at time $t= 0$ is denoted as ${\mathbf {X}}_0$. In a small-strain framework the motion of a material point over time can be described by

1 $$\begin{aligned} {\mathbf {X}}= {\mathbf {X}}_0 + {\mathbf {u}}\end{aligned}$$

in which ${\mathbf {u}}= {\mathbf {u}}({\mathbf {X}}_0, t)$ is the displacement vector. Assuming small-strain kinematics, the strain at the macroscale, ${\mathsf {\varepsilon }}_\mathrm{M}= {\mathsf {\varepsilon }}_\mathrm{M}({\mathbf {X}}_0, t)$ is then defined as

2 $$\begin{aligned} {\mathsf {\varepsilon }}_\mathrm{M}= \mathbf {\nabla }^\mathrm{s}{\mathbf {u}}\end{aligned}$$

Static equilibrium at the macroscale implies

3 $$\begin{aligned} \mathbf {\nabla }\cdot {\mathsf {\sigma }}_\mathrm{M}+ \mathbf {b}&= 0 \end{aligned}$$

where the stress tensor ${\mathsf {\sigma }}_\mathrm{M}({\mathsf {\varepsilon }}_\mathrm{M}, {\mathbf {\xi }})$ depends on the strain tensor ${\mathsf {\varepsilon }}_\mathrm{M}$ and the history parameters ${\mathbf {\xi }}$. The body forces are denoted by $\mathbf {b}$. The macroscopic stress ${\mathsf {\sigma }}_\mathrm{M}$ is obtained from the RVE problem through the micro-macroscale transition.

### Microscale problem

To characterize the microstructure of the material a Representative Volume Element $\mathcal {V}_\mathrm{m}$ is defined. The position of a material point in the RVE over time is described by ${\mathbf {x}}(t)$. The reference configuration at $t= 0$ is denoted by ${\mathbf {x}}_0$. The displacement of a material point over time is a superposition of the displacement induced by the macroscopic deformation ${\mathsf {\varepsilon }}_\mathrm{M}\cdot {\mathbf {x}}_0$ and the microscopic fluctuation ${\mathbf {w}}= {\mathbf {w}}({\mathbf {x}}_0, t)$,

4 $$\begin{aligned} {\mathbf {x}}(t)&= {\mathbf {x}}_0 + {\mathsf {\varepsilon }}_\mathrm{M}\cdot {\mathbf {x}}_0 + {\mathbf {w}}({\mathbf {x}}_0, t)&\mathrm{in} ~ \mathcal {V}_\mathrm{m}\end{aligned}$$

The microscopic strain ${\mathsf {\varepsilon }}_\mathrm{m}$ is then given by

5 $$\begin{aligned} {\mathsf {\varepsilon }}_\mathrm{m}&= {\mathsf {\varepsilon }}_\mathrm{M}+ \mathbf {\nabla }^\mathrm{s}{\mathbf {w}}&\mathrm{in} ~ \mathcal {V}_\mathrm{m}\end{aligned}$$

The influence of the body forces is assumed to be negligible at this scale, such that the linear momentum balance of the RVE simplifies to

6 $$\begin{aligned} \mathbf {\nabla }\cdot {\mathsf {\sigma }}_\mathrm{m}&= \mathbf {0}&\mathrm{in} ~ \mathcal {V}_\mathrm{m} \end{aligned}$$

where ${\mathsf {\sigma }}_\mathrm{m}= {\mathsf {\sigma }}_\mathrm{m}({\mathsf {\varepsilon }}_\mathrm{m}, {\mathbf {\xi }})$ is the microscopic Cauchy stress and ${\mathbf {\xi }}$ are the history variables required for the constitutive relation.

### Coupling of the scales

The homogenization is based on two principles. The first principle is the volume average of the stress or the strain, which should match the macroscopic strain and stress respectively.

$$\begin{aligned} \varvec{{\mathsf {\varepsilon }}}_\mathrm{M}&= \frac{1}{|\mathcal {V}_\mathrm{m}|} \int _{\mathcal {V}_\mathrm{m}} \varvec{{\mathsf {\varepsilon }}}_\mathrm{m}\, \mathrm{d}{\mathcal {V}_\mathrm{m}} \end{aligned}$$

The second principle is the Hill–Mandel condition [43] which prescribes that the virtual work performed per unit volume at the macroscale should equal the volume average of the virtual work at the microscale.

$$\begin{aligned} {\mathsf {\sigma }}_\mathrm{M}: \delta {\mathsf {\varepsilon }}_\mathrm{M}&= \frac{1}{|\mathcal {V}_\mathrm{m}|} \int _{\mathcal {V}_\mathrm{m}} \varvec{{\mathsf {\sigma }}}_\mathrm{m}: \delta {\mathsf {\varepsilon }}_\mathrm{m}\, \mathrm{d}{\mathcal {V}_\mathrm{m}} \end{aligned}$$

It has been shown that periodic boundary conditions on the microfluctuation field ${\mathbf {w}}^+ = {\mathbf {w}}^-$ comply with these homogenization equations. To discriminate rigid body displacements and rotations, the microfluctuations on the corners of the RVE $\mathcal {S}_\mathrm{m}$ are constrained by ${\mathbf {w}}= \mathbf {0}$. The macroscopic stress ${\mathsf {\sigma }}_\mathrm{M}$ is then given by volume averaging the microscopic stress ${\mathsf {\sigma }}_\mathrm{m}$ and the macroscopic tangent stiffness can be deduced using, for example, direct condensation [44].

### Spatial discretization

Both the macroscopic and microscopic problems, Eqs. (3) and (6) can be put in the weak form by following the Bubnov–Galerkin approach. Integrating these equations with test-functions ${\mathbf {\phi }}_\mathrm{M}({\mathbf {X}}_0)$ and ${\mathbf {\phi }}_\mathrm{m}({\mathbf {x}}_0)$ respectively and application of the divergence theorem, yields:

7 $$\begin{aligned} \int _{\mathcal {V}_\mathrm{M}} \mathbf {\nabla }^\mathrm{s}{\mathbf {\phi }}_\mathrm{M}: {\mathsf {\sigma }}_\mathrm{M}({\mathsf {\varepsilon }}_\mathrm{M}) \, \mathrm{d}{\mathcal {V}_\mathrm{M}}&= \int _{\mathcal {S}_\mathrm{M}} {\mathbf {\phi }}_\mathrm{M}\cdot \mathbf {t}\, \mathrm{d}{\mathcal {S}_\mathrm{M}}&\text { for all } {\mathbf {\phi }}_\mathrm{M}\end{aligned}$$

8 $$\begin{aligned} \int _{\mathcal {V}_\mathrm{m}} \mathbf {\nabla }^\mathrm{s}{\mathbf {\phi }}_\mathrm{m}: {\mathsf {\sigma }}_\mathrm{m}({\mathbf {x}}, {\mathbf {\xi }}) \, \mathrm{d}{\mathcal {V}_\mathrm{m}}&= 0&\text { for all } {\mathbf {\phi }}_\mathrm{m}\end{aligned}$$

Both the macro- and microscopic domain are discretized using standard, Lagrangian finite elements. The discretization introduces interpolation functions $\mathbf {N}_i({\mathbf {X}}_0)$, and nodes for $i=1,\ldots ,n^\mathrm{d}_\mathrm{M}$ at the macroscale and $\mathbf {N}_j({\mathbf {x}}_0)$ for $j=1,\ldots ,n^\mathrm{d}_\mathrm{m}$ at the microscale. Here, $n^\mathrm{d}_\mathrm{M}$ and $n^\mathrm{d}_\mathrm{m}$ denote the number of nodes and thereby implicitly define the number of unknowns.

The trial functions ${\mathbf {\phi }}_\mathrm{M}({\mathbf {X}}_0)$ and ${\mathbf {\phi }}_\mathrm{m}({\mathbf {x}}_0)$ are approximated by their discrete counterparts ${\mathbf {\phi }}_\mathrm{M}({\mathbf {X}}_0) \approx {\mathbf {\phi }}^{h}_\mathrm{M}({\mathbf {X}}_0)$, ${\mathbf {\phi }}_\mathrm{m}({\mathbf {x}}_0) \approx {\mathbf {\phi }}^{h}_\mathrm{m}({\mathbf {x}}_0)$. A similar approximation is used for the displacement-field ${\mathbf {u}}({\mathbf {X}}_0) \approx {\mathbf {u}}^{h}({\mathbf {X}}_0)$ and the microfluctuation field ${\mathbf {w}}({\mathbf {x}}_0) \approx {\mathbf {w}}^{h}({\mathbf {x}}_0)$.

$$\begin{aligned}&{\mathbf {\phi }}^{h}_\mathrm{M}({\mathbf {X}}_0) = \sum _i { {\phi } } _{\mathrm{M}i} \mathbf {N}_i({\mathbf {X}}_0)&{\mathbf {u}}^{h}({\mathbf {X}}_0, t) = \sum _j { {u} } _j(t) \mathbf {N}_j({\mathbf {X}}_0) \\&{\mathbf {\phi }}^{h}_\mathrm{m}({\mathbf {x}}_0) = \sum _i { {\phi } } _{\mathrm{m}i} \mathbf {N}_i({\mathbf {x}}_0)&{\mathbf {w}}^{h}({\mathbf {x}}_0, t) = \sum _j { {w} } _j(t) \mathbf {N}_j({\mathbf {x}}_0) \end{aligned}$$

where ${\mathbf {w}}^{h}({\mathbf {x}}_0)$ is constrained using periodic boundary conditions on the RVE’s boundary and vanishing at the corners. The macroscopic problem takes conventional boundary conditions, e.g. displacements ${\mathbf {u}}^{h} = {\mathbf {u}}^*$ or tractions ${\mathsf {\sigma }}_\mathrm{M}\cdot \mathbf {n}= \mathbf {t}^*$. The Newton-Raphson method is used to solve the resulting equilibrium Eqs. (7) and (8).

### Computational size and cost of the problem

Applying computational homogenization to a high-fidelity problem with ample microscopic details entails a significant increase of the required time and computational resources. Despite the reductions achieved by the homogenization procedures, the size of the macroscopic problem that can be computed within a feasible amount of time and computational resources is still limited for RVEs with nonlinear constituents.

The term FE${}^2$ by Chaboche illustrates the computational difficulty of the homogenization problem. An RVE needs to be resolved in every integration point at the macroscale. The amount of iterations required to solve the momentum balance on the macro- and the microscale is denoted by $n^\mathrm{it}_\mathrm{M}$ and $n^\mathrm{it}_\mathrm{m}$ respectively. The number of Degrees of Freedom (DOF) on the macro- and the microscale are denoted by $n^\mathrm{d}_\mathrm{M}$ and $n^\mathrm{d}_\mathrm{m}$ respectively. For a nonlinear RVE, the required amount of floating point operations (FLOPs) to solve the matrix-vector equations are given by

$$\begin{aligned} \#^\mathrm{flop}_\mathrm{LA}&\propto \mathcal {O}\left( {n^\mathrm{it}_\mathrm{M}}\right) \times \mathcal {O}\left( {n^\mathrm{d}_\mathrm{M}\log (n^\mathrm{d}_\mathrm{M})}\right) \\&+ \mathcal {O}\left( {n^\mathrm{g}_\mathrm{M}}\right) \times \mathcal {O}\left( {n^\mathrm{it}_\mathrm{M}}\right) \times \mathcal {O}\left( {n^\mathrm{it}_\mathrm{m}}\right) \times \mathcal {O}\left( {n^\mathrm{d}_\mathrm{m}\log (n^\mathrm{d}_\mathrm{m})}\right) \end{aligned}$$

in which $\#^\mathrm{flop}_\mathrm{LA}$ is the number of required FLOPs to solve the matrix-vector systems in the homogenization problem.

The same scaling relation of the number of floating point operations holds for the number of evaluations of the ordinary differential equation in the material model. The ordinary differential equation in the material model has to be solved in every integration point at the microscale. The number of integration points on the macro- and microscale are denoted by $n^\mathrm{g}_\mathrm{M}$ and $n^\mathrm{g}_\mathrm{m}$ respectively. The number of FLOPs associated with solving the material model is proportional to the number of integration points and iterations

$$\begin{aligned} \#^\mathrm{flop}_\mathrm{mat}\propto \mathcal {O}\left( {n^\mathrm{it}_\mathrm{M}}\right) \times \mathcal {O}\left( {n^\mathrm{g}_\mathrm{M}}\right) \times \mathcal {O}\left( {n^\mathrm{it}_\mathrm{m}}\right) \times \mathcal {O}\left( {n^\mathrm{g}_\mathrm{m}}\right) \end{aligned}$$

in which $\#^\mathrm{flop}_\mathrm{mat}$ is the number of required FLOPs to solve the material models used in the RVE. The total number of calculations is then given by

$$\begin{aligned} \#^\mathrm{flop}\propto \#^\mathrm{flop}_\mathrm{LA}+ \#^\mathrm{flop}_\mathrm{mat}\end{aligned}$$

The computational costs associated with FE${}^2$ problems are quantitatively illustrated using a straightforward example. Let’s consider a micro-structural element that is discretized with a grid of $100 \times 100$ quadrilateral elements with 4 integration points per element, yielding $n^\mathrm{g}_\mathrm{m}\approx 40000$ and $n^\mathrm{d}\approx 80000$. When the macroscopic problem is of the same dimension and an assumed average of 4 iterations in the Newton-Raphson procedures, the number of FLOPs per time increment required to solve the matrix-vector systems $\#^\mathrm{flop}_\mathrm{LA}$ is in the order of $\mathcal {O}\left( {10^{11}}\right)$ and the number of FLOPs associated with solving the nonlinear material model $\#^\mathrm{flop}_\mathrm{mat}$ is in the order of $\mathcal {O}\left( {10^{10}}\right)$.

## Reduced order modeling

As shown in the previous section, the computational cost of the homogenization scales with the number of DOFs $n^\mathrm{d}_\mathrm{M}$ and $n^\mathrm{d}_\mathrm{m}$ and the number of integration points $n^\mathrm{g}_\mathrm{M}$ and $n^\mathrm{g}_\mathrm{m}$. By reducing the amount of DOFs of the RVE problem, the costs of solving the matrix-vector equations in the computational homogenization problem will decrease proportionally.

Using a reduced basis, the finite element discretization is mapped onto a global basis. In most physical problems this yields a significant reduction of the required number of DOFs $n^\mathrm{d}_\mathrm{m}$ since the problem will have considerably less physical modes for the kinematics then the number of local basis functions required to capture all the local features. Reduced Order Modeling using the Reduced Basis technique is outlined in Sect. 3.1.

After the reduction of the number of DOFs the number of integration points $n^\mathrm{g}_\mathrm{m}$ is still equivalent to the original problem. The reduction of the integral is done using High-Performance Reduced Order Modeling or Empirical Cubature, outlined in Sect. 4.

### Reduced basis approach

The number of DOFs $n^\mathrm{d}$ scales with the spatial resolution of the mesh. The number of kinematic modes $n^{w}$ present in the RVE is independent of the spatial resolution. Moreover, many modes have a negligible contribution. When discretizing the RVE, without prior knowledge, the number of DOFs used to accurately capture these kinematics is relatively high with respect to the relevant number of relevant modes.

If there is prior knowledge of the kinematics occurring in the RVE, it is possible to construct a basis that captures the kinematics more efficiently with a limited number of DOFs. Since Computational Homogenization often involves repetitive calculations of an RVE, it calls for an additional optimization step. The extra computational costs for identifying a Reduced Basis for the problem are justified by the higher costs for solving the RVE multiple times.

To find a more optimal basis for the microscale problem, prior knowledge of the relevant kinematics needs to be obtained. This is done by repetitively solving the RVE-problem under different macroscopic strains ${\mathsf {\varepsilon }}^i_\mathrm{M}(t_j)$ for $i = 1,\ldots , n^\mathrm{\ell }$ and $j = 1,\ldots , n^\mathrm{t}$ to gather a database with snapshots of the occurring microfluctuation fields. Here, each separate time-step of each load-case is considered as a snapshot, which is numbered as *k*. In total there are $n^\mathrm{s}= n^\mathrm{\ell }\cdot n^\mathrm{t}$ snapshots of the microfluctuation field. The snapshot of the microfluctuation field ${\mathbf {w}}^{h}_k({\mathbf {x}}_0)$ is then expressed by

9 $$\begin{aligned} {\mathbf {w}}^{h}_k({\mathbf {x}}_0)&= { {w} } _{kl} \mathbf {N}_l({\mathbf {x}}_0)&\text {for } k = 1,\ldots , n^\mathrm{s}, \, l = 1,\ldots , n^\mathrm{d}\end{aligned}$$

Equation (9) can be rewritten as a matrix-vector equation where the snapshots are collected in the so-called *snapshot-matrix* $\underline{\underline{\mathfrak {X}}} \in \mathbb {R}^{n^\mathrm{s}\times n^\mathrm{d}}$ in which $\left. \underline{\underline{\mathfrak {X}}} \right| _{kl} = { {w} } _{kl}$.

An orthonormal basis is derived from the snapshot matrix $\underline{\underline{\mathfrak {X}}}$ using the Proper Orthogonal Decomposition (POD). This mathematical procedure provides the most optimal basis (in the least-square sense) to represent the snapshots in combination with their corresponding eigenvalue $\lambda _i$ 1. The eigenvalues denote the energy-content of their corresponding eigenmodes $\underline{v}_i$ in representing the modes of the DOF.

**Fig. 2:**
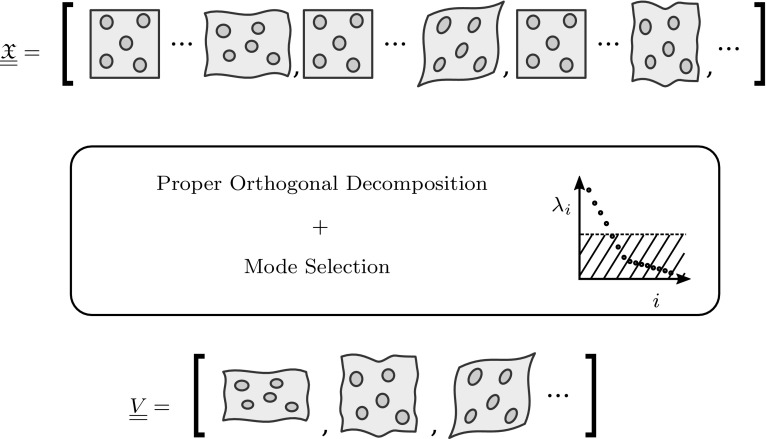
Outline of the proper orthogonal decomposition

Based on their eigenvalues the most important modes can be selected to construct a Reduced Basis for the microscale problem. The eigenvalues and their corresponding eigenvectors are sorted in descending order, allowing to construct the basis from the first $n^{w}$ eigenvectors. The required number of modes to capture the snapshot up to a given tolerance $\delta \in [0,1]$ is found by increasing $n^{w}$ until the following relation holds^2^

10 $$\begin{aligned} 1 - \frac{\sum ^{n^{w}}_{i=1} \lambda _i }{\sum ^{n^\mathrm{s}}_{i=1} \lambda _i} \le \delta \end{aligned}$$

The Proper Orthogonal Decomposition is outlined schematically in Fig. 2.

### Construction of the reduced order model

After determining the number of required modes $\underline{\underline{V}} = [ \underline{v}_1,\underline{v}_2, \ldots , \underline{v}_{n^{w}}]$ the reduced basis functions $\mathbf {R}_l({\mathbf {x}}_0)$ can be constructed as a linear combination of the traditional basis functions $\mathbf {N}_m({\mathbf {x}}_0)$ using the modes $\underline{v}_l$ resulting from the POD.

11 $$\begin{aligned} \mathbf {R}_l({\mathbf {x}}_0)&= \sum _m V_{lm} \mathbf {N}_m({\mathbf {x}}_0) \end{aligned}$$

Using a Galerkin approach for discretizing the reduced system, the test and trial functions are discretized using the reduced basis

$$\begin{aligned} {\mathbf {\phi }}^{h}({\mathbf {x}}_0, t)= & {} \sum _i {\varPhi }_i(t) \mathbf {R}_i({\mathbf {x}}_0)\\ {\mathbf {w}}^{h}({\mathbf {x}}_0, t)= & {} \sum _j {W}_j(t) \mathbf {R}_j({\mathbf {x}}_0) \end{aligned}$$

which ${\varPhi }_j$ and ${W}_i$ are the reduced test and microfluctuation coefficients. Substituting the reduced functions into the weak formulation of the microscopic momentum balance (8) the following reduced internal force $\bar{f}^\mathrm{int}$ is found

12 $$\begin{aligned} \bar{f}^\mathrm{int}_i({\mathbf {w}}^{h})&= - \int _{\mathcal {V}_\mathrm{m}} \mathbf {\nabla }^\mathrm{s}\mathbf {R}_i({\mathbf {x}}_0) : {\mathsf {\sigma }}_\mathrm{m}({\mathsf {\varepsilon }}_\mathrm{M}, \underline{{W}}, {\mathbf {\xi }}) \, \mathrm{d}{\mathcal {V}_\mathrm{m}} \end{aligned}$$

13 $$\begin{aligned}&= - V_{il} \int _{\mathcal {V}_\mathrm{m}} \mathbf {\nabla }^\mathrm{s}\mathbf {N}_l({\mathbf {x}}_0) : {\mathsf {\sigma }}_\mathrm{m}({\mathsf {\varepsilon }}_\mathrm{M}, \underline{{W}}, {\mathbf {\xi }}) \, \mathrm{d}{\mathcal {V}_\mathrm{m}} \end{aligned}$$

with $i = 1,\ldots , n^{w}$ and $\underline{{W}}$ the reduced vector with unknowns $[{W}_1, {W}_2, \ldots , {W}_{n^{w}}]^T$ used to discretize the microfluctuation field. The number of modes $n^{w}$ is often orders of magnitude smaller than the number of DOFs $n^\mathrm{d}$. The linear momentum balance is solved using the same Newton-Raphson procedure for the microscale problem. The computational costs for solving the system of equations scales proportionally to the number of microfluctuation modes $n^{w}$.

$$\begin{aligned} \#^\mathrm{flop}_\mathrm{LA}&\propto \mathcal {O}\left( {n^\mathrm{it}_\mathrm{M}}\right) \times \mathcal {O}\left( {n^\mathrm{d}_\mathrm{M}\log (n^\mathrm{d}_\mathrm{M})}\right) \\&+ \mathcal {O}\left( {n^\mathrm{g}_\mathrm{M}}\right) \times \mathcal {O}\left( {n^\mathrm{it}_\mathrm{M}}\right) \times \mathcal {O}\left( {n^\mathrm{it}_\mathrm{m}}\right) \times \mathcal {O}\left( {n^{w}\log (n^{w})}\right) \end{aligned}$$

After reducing the number of microfluctuation DOFs, the computational costs for solving the RVE problem are determined by the solution of the material-model. Due to the nonlinear character of the internal force, the integral in equation (13) can not be precomputed. This procedure remains computationally expensive since the material model needs to be resolved on each integration point to compute the integral of the internal force.

## High-performance reduced order modeling

In this section two approaches to reduce the costs of evaluating the nonlinear internal force are reviewed:

1. The Empirical Interpolation Method (EIM) [42] projects the nonlinear term onto a reduced basis for the stress field. The stress field is approximated by the reduced modal basis, enabling pre-computation of the integral.
2. Alternatively, the integration scheme can be reduced directly. This approach is known in literature as Energy-Conserving Sampling and Weighting (ECSW) hyper reduction method or Empirical Cubature Method (ECM) [33]. The integration costs are reduced by using a preselected subset of sampling points and assigning a positive weighting factor to them.

### Empirical interpolation method

In the Empirical Interpolation Method, the stress field is approximated by interpolating between weighted stress modes $\varPsi ^j({\mathbf {x}}_0)$ dependent on the microscopic coordinates, which are scaled by the coefficients $C^j({\mathsf {\varepsilon }}_\mathrm{M}, {\mathbf {\xi }})$ that depend on the macroscopic deformation ${\mathsf {\varepsilon }}_\mathrm{M}$ and material point history ${\mathbf {\xi }}$. The advantage of introducing modes for the stress-field is that the integration of these modes can be performed in the off-line stage since the interpolating coefficients no longer have a spatial dependency.

The weighted strain modes $\varPhi ^k({\mathbf {x}}_g)$ are constructed by taking the symmetric gradient of the micro-fluctuation modes weighted by the square-root of the integration weight (including the Jacobian). The weighted stress modes ${\mathsf {\mu }}^k({\mathbf {x}}_g)$ are obtained from the square-root weighted stress snapshot matrix $\underline{\underline{\mathfrak {X}}}_{{\mathsf {\sigma }}}$. The orthogonal weighted stress-basis can be obtained by applying a standard POD procedure on the vectorized components of the sampled stresses or by using a tensorial decomposition such as the procedure outlined by Roussette et al. [45].

14 $$\begin{aligned} \varPhi ^k({\mathbf {x}}_g)&= \sqrt{w_g} \mathbf {\nabla }^\mathrm{s}\mathbf {R}^k({\mathbf {x}}_g) \end{aligned}$$

15 $$\begin{aligned} \varPsi ^k({\mathbf {x}}_g)&= {\mathsf {\mu }}^k({\mathbf {x}}_g) \end{aligned}$$

The square-root weighted stress and strain can be approximated as

16 $$\begin{aligned} \sqrt{w_g} {\mathsf {\varepsilon }}_\mathrm{m}({\mathbf {x}}_g)&\approx \sum _i \varPhi ^i({\mathbf {x}}_g) { {W} } _i \end{aligned}$$

17 $$\begin{aligned} \sqrt{w_g} {\mathsf {\sigma }}_\mathrm{m}({\mathbf {x}}_g, {\mathbf {\xi }}, {\mathsf {\varepsilon }}_\mathrm{M})&\approx \sum _j \varPsi ^j({\mathbf {x}}_g) C^j({\mathsf {\varepsilon }}_\mathrm{M}, {\mathbf {\xi }}) \end{aligned}$$

Substituting the stress basis (14) into the reduced order model (12) after applying Gaussian quadrature to calculate the integral leads to the following expressions for the internal force vector.

18 $$\begin{aligned} \bar{f}^\mathrm{int}_i({\mathbf {w}}^{h}) = -\sum _{g=1}^{n^\mathrm{g}} \varPhi ^i({\mathbf {x}}_g) \varPsi ^j({\mathbf {x}}_{\mathrm{g}}) \, C^j({\mathsf {\varepsilon }}_\mathrm{M}, {\mathbf {\xi }}) \end{aligned}$$

The modal coefficients $C^j({\mathsf {\varepsilon }}_\mathrm{M}, {\mathbf {\xi }})$ are to still be determined for the current strain state. The stress modes are weighted by selecting a set of integration points as sampling points and applying a Least-Square fitting such that the error between these points and the approximated stresses will be minimized in the Least-Squares sense.

#### Reformulation of the ill-posed stress–strain problem

The stress- and strain basis are composed out of stress- and strain-fields resulting from the converged linear momentum balance (8). This leads to an ill-posed system of equations since the reduced linear momentum balance (18) constructed using the modal bases for the stress- and strain fields is in equilibrium regardless of the coefficients $\underline{C}_{{\mathsf {\sigma }}}$, as stated in [42].

In order to solve this problem, the Expanded Basis Approach (EBA) is applied, where the stress-basis $\varPsi$ and corresponding coefficients $\underline{C}_{{\mathsf {\sigma }}}$ are enriched with the weighted strain basis $\varPhi$ and corresponding (inadmissible) coefficients $\underline{C}_{{\mathsf {\varepsilon }}}$, such that the basis does not satisfy equilibrium independently of the coefficients $\underline{C}_{{\mathsf {\sigma }}}$.

19 $$\begin{aligned} \varPsi ^{\mathrm{ex}} = \varPsi \oplus \varPhi \end{aligned}$$

By rewriting the strains and stresses in Voigt notation, the tensorial bases $\varPsi$ and $\varPhi$ are reformatted into the matrix formats $\underline{\underline{\varPsi }}$ and $\underline{\underline{\varPhi }}$ respectively. An example of this procedure is illustrated for a set of two-dimensional strain modes:

$$\begin{aligned} \underline{\underline{\varPhi }} = \left[ \begin{array}{cccc} \varPhi _1({\mathbf {x}}_1)|_{xx} &{} \varPhi _2({\mathbf {x}}_1)|_{xx} &{} \ldots &{} \varPhi _{n^{w}}({\mathbf {x}}_1)|_{xx} \\ \varPhi _1({\mathbf {x}}_1)|_{yy} &{} \varPhi _2({\mathbf {x}}_1)|_{yy} &{} \ldots &{} \varPhi _{n^{w}}({\mathbf {x}}_1)|_{yy} \\ 2\varPhi _1({\mathbf {x}}_1)|_{xy} &{} 2\varPhi _2({\mathbf {x}}_1)|_{xy} &{} \ldots &{} 2\varPhi _{n^{w}}({\mathbf {x}}_1)|_{xy} \\ \varPhi _1({\mathbf {x}}_2)|_{xx} &{} \varPhi _2({\mathbf {x}}_2)|_{xx} &{} \ldots &{} \varPhi _{n^{w}}({\mathbf {x}}_2)|_{xx} \\ \vdots &{} \vdots &{} &{} \vdots \\ 2\varPhi _1({\mathbf {x}}_{n^\mathrm{g}})|_{xy} &{} 2\varPhi _2({\mathbf {x}}_{n^\mathrm{g}})|_{xy} &{} \ldots &{} 2\varPhi _{n^{w}}({\mathbf {x}}_{n^\mathrm{g}})|_{xy} \\ \end{array} \right] \end{aligned}$$

The gappy stress basis $\hat{\underline{\underline{\varPsi }}}$ is formed by selecting all the rows corresponding to the selected integration points ${\mathbf {x}}_g$ for $g \in \mathcal {I}$ where $\mathcal {I}$ is the set with selected integration point indices.

The coefficients for the expanded basis (19) $\underline{C} = [ \underline{C}^T_{{\mathsf {\sigma }}}, \underline{C}^T_{{\mathsf {\varepsilon }}} ]^T$ are identified from a set of $\hat{n}^\mathrm{g}\ll n^\mathrm{g}$ sampled *gappy stresses* in Voigt notation $\hat{\underline{ { \sigma } }}$ to approximate the stress in the Least-Squares sense using:

20 $$\begin{aligned} \left( \begin{array}{c} \underline{C}_{ { \sigma } } \\ \underline{C}_{ { \varepsilon } } \\ \end{array} \right) = {\underbrace{ \left[ \begin{array}{cc} \hat{\underline{\underline{\varPsi }}}^T \hat{\underline{\underline{\varPsi }}} &{} \hat{\underline{\underline{\varPsi }}}^T \hat{\underline{\underline{\varPhi }}} \\ \hat{\underline{\underline{\varPhi }}}^T \hat{\underline{\underline{\varPsi }}} &{} \hat{\underline{\underline{\varPhi }}}^T \hat{\underline{\underline{\varPhi }}} \\ \end{array} \right] }_{\underline{\underline{\hat{M}}}} }^{-1} \left[ \begin{array}{cc} \hat{\underline{\underline{\varPsi }}}^T \\ \hat{\underline{\underline{\varPhi }}}^T \\ \end{array} \right] \hat{\underline{ { \sigma } }} \end{aligned}$$

Using the Schur complement, the coefficients $\underline{C}$ can be expressed by

21 $$\begin{aligned} \underline{C}_{ { \sigma } }&= \hat{\underline{\underline{\varPsi }}}^\dag \left( \hat{\underline{\underline{{\mathsf {\sigma }}}}} - \hat{\underline{\underline{\varPhi }}} \underline{C}_{ { \varepsilon } }\right) \end{aligned}$$

22 $$\begin{aligned} \underline{C}_{ { \varepsilon } }&= \underline{\underline{S}}^{-1} \hat{\underline{\underline{\varPhi }}}^T \left( \underline{\underline{I}}- \hat{\underline{\underline{\varPsi }}} \hat{\underline{\underline{\varPsi }}}^\dag \right) \hat{\underline{ { \sigma } }} \end{aligned}$$

in which the matrix $\hat{\underline{\underline{\varPsi }}}^\dag$ is the pseudo-inverse of the gappy-stress basis matrix and $\underline{\underline{S}}$ is Schur’s complement matrix defined by

23 $$\begin{aligned} \hat{\underline{\underline{\varPsi }}}^\dag&= \left( \hat{\underline{\underline{\varPsi }}}^T \hat{\underline{\underline{\varPsi }}} \right) ^{-1} \hat{\underline{\underline{\varPsi }}} \end{aligned}$$

24 $$\begin{aligned} \underline{\underline{S}}&= \hat{\underline{\underline{\varPhi }}}^T \left( \underline{\underline{I}}- \hat{\underline{\underline{\varPsi }}} \hat{\underline{\underline{\varPsi }}}^\dag \right) \end{aligned}$$

Matrix $\underline{\underline{S}}$ is invertible since the sampled stressed are chosen such that $\hat{\varPsi }^{\mathrm{ex}}$ is of full rank. The problem can be rewritten into finding a solution for which the inadmissible coefficients vanish, i.e. $\underline{C}_{ { \varepsilon } } = \underline{0}$, such that the stress solution is interpolated using only stress basis vectors in equilibrium. Since $\underline{\underline{S}}$ is non-singular, as shown by [42], the coefficients can only be $\underline{0}$ when the following holds:

25 $$\begin{aligned} \underbrace{\hat{\underline{\underline{\varPhi }}}^T \left( \underline{\underline{I}}- \hat{\underline{\underline{\varPsi }}} \hat{\underline{\underline{\varPsi }}}^\dag \right) }_{\underline{\underline{\varPhi }}^*} \hat{\underline{ { \sigma } }}(\underline{{W}}, {\mathsf {\varepsilon }}_\mathrm{M}) = \underline{0} \end{aligned}$$

This form of the problem is referred to as the *hyper-reduced* problem [42].

#### Gappy point selection

To complete the hyper-reduced model a set of integration points ${\mathbf {x}}_g$ for which $g \in \mathcal {I}$ suitable for the Empirical Interpolation of the stress field is chosen. This is done by using the snapshots of the stress field as samples to find a set of integration points that yield a good approximation of the complete stress-field. It is computationally intractable to evaluate the approximative qualities of the subset of integration points for all $\genfrac(){0.0pt}1{n^\mathrm{g}}{\hat{n}^\mathrm{g}}$ possible combinations. Therefore the sub-optimal Greedy algorithm is applied to find a set of integration points which has a good interpolating quality for the snapshot data [42].

To improve the stability of the system of equations, the selected points are complemented with a second set of points selected from the remaining integration points. These points are selected using the Greedy algorithm using a criterion that aims at optimizing the conditioning of the resulting reduced tangent stiffness matrix.

*Accuracy points* Hernández et al. [42] select the integration points based on minimizing the error between the stress snapshots ${\mathsf {\sigma }}^j$ and the interpolated stress ${\mathsf {\sigma }}^*_j(\varPsi , \mathcal {I})$ constructed using the subset of integration points ${\mathbf {x}}_g$ with $g \in \mathcal {I}$. The error in the stress approximation is given by

26 $$\begin{aligned} \epsilon (\varPsi , \mathcal {I}) = \sqrt{\sum _{j=1}^{n^\mathrm{s}} \Vert {\mathsf {\sigma }}_j - {\mathsf {\sigma }}^*_j(\varPsi , \mathcal {I}) \Vert ^2} \end{aligned}$$

which is split up by Hernández et al. [42] into a *truncation error* that represents the error introduced due to the use of a reduced stress basis and a *reconstruction error* $\epsilon ^\mathrm{rec}$. The reconstruction error measures the error introduced by the Least-Squares fit of the stresses onto the modal basis, which is given by

27 $$\begin{aligned} \epsilon ^\mathrm{rec}&= \frac{1}{|\mathcal {V}_\mathrm{m}|} \Vert \underline{\underline{\varPsi }} \underline{\underline{\varPsi }}^T \underline{\underline{\mathfrak {X}}} - \underline{\underline{R}}(\mathcal {I}) \underline{\underline{\mathfrak {X}}}_{\mathcal {I}} \Vert _\mathrm {F}\end{aligned}$$

This error measure is used to select the integration points that are best suited for an accurate stress interpolation.

*Stability points* Hernández reported that using integration points that are only selected on the basis of accuracy, provides a system of equations that is not unconditionally stable. Therefore, the set of selected integration points needs to be complemented with a set of extra points to ensure the stability of the system. The same Greedy selection procedure is used with a criterion that aims to optimize the positive-definiteness of the tangent stiffness matrix. To ensure maximum stability, the criterion given in [42] that needs to be minimized reads

28 $$\begin{aligned} \mathrm {cond}(K) \propto \frac{\Vert \hat{\underline{\underline{R}}} \hat{\varPhi } \Vert _\mathrm {F}}{\Vert (\underline{\underline{I}}- \hat{\underline{\underline{R}}})\hat{\varPhi } \Vert _\mathrm {F}} \end{aligned}$$

This yields a second set of integration points in favor of the stability of the Empirical Stress Interpolation.

#### Reconstruction of the reduced stress field

The resulting weighted stress field in the RVE is given by the Empirical Interpolation (17). The coefficients $\underline{C}_{ { \sigma } }$ can be found using the expression (21) by substituting $\underline{C}_{ { \varepsilon } } = 0$.

29 $$\begin{aligned} \underline{ { \sigma } }({\mathbf {x}}_g, t) = \underbrace{\underline{\underline{\varPsi }} \hat{\underline{\underline{\varPsi }}}^\dag }_{\underline{\underline{R}}} \hat{\underline{ { \sigma } }}(\hat{{\mathbf {x}}}_g, t). \end{aligned}$$

where $\underline{\underline{R}}$ is referred to as the *weighted reconstruction matrix*.

To reconstruct the macroscopic stress, the reconstructed stress-field ${\mathsf {\sigma }}_\mathrm{m}({\mathbf {x}}_g, t)$ has to be integrated over the RVE and volume averaged. The relation between the gappy-stress and the macroscopic stress is then given by the following linear operator:

30 $$\begin{aligned} \underline{\underline{T}}&= \frac{1}{|\mathcal {V}_\mathrm{m}|} \left[ \sqrt{w_1} \underline{\underline{I}}, \sqrt{w_2} \underline{\underline{I}}, \ldots \sqrt{w_n^\mathrm{g}} \underline{\underline{I}}\right] \underline{\underline{R}}\end{aligned}$$

which projects the gappy stresses $\hat{\underline{ { \sigma } }}$ onto the macroscopic stress $\underline{ { \sigma } }_\mathrm{M}= \underline{\underline{T}} \hat{\underline{ { \sigma } }}$.

### Empirical cubature method

Instead of approximating the stress field using a Proper Orthogonal Basis constructed from the stress snapshots, the expensive integral of the internal forces can be approximated using the Empirical Cubature Method.

For a polynomial FEM basis one can construct an exact quadrature scheme. This classical Gaussian quadrature scheme approximates the integral summing up weighted samples of the integrand at specific points, the Gauss integration points

31 $$\begin{aligned} f_i^\mathrm{int}&= \int _{\mathcal {V}_\mathrm{m}} \mathbf {\nabla }^\mathrm{s}\mathbf {N}({\mathbf {x}}) : {\mathsf {\sigma }}({\mathbf {x}}) \, \mathrm{d}{\mathcal {V}_\mathrm{m}} \approx \sum _{g=1}^{n^\mathrm{g}} w_g \mathbf {\nabla }^\mathrm{s}\mathbf {N}({\mathbf {x}}_g) : {\mathsf {\sigma }}({\mathbf {x}}_g) \end{aligned}$$

where $w_g$ denote the Gaussian quadrature weights (including the determinant of the Jacobian).

When rewriting the problem using the reduced-order basis to (12) one can imagine that it is possible to reduce the integration as well since the number of unknowns describing the integrand has decreased drastically. The underlying idea of the reduced integration is to select a subset of integration points and appoint a (positive) weight to them such that they approximate the integral as accurately as possible. This concept is demonstrated in [33, 40, 41].

The method will be outlined briefly in two steps. First the determination of the integration weights is discussed; next the selection procedure for the integration points is presented.

#### Determining the integration weights

To determine the weights, the internal force contributions at each integration point $\mathbf {f}_i^p({\mathbf {x}}_g)$ under snapshot $p = 1,\ldots , n^\mathrm{s}$ resulting from each modal virtual strain $\mathbf {\nabla }^\mathrm{s}\mathbf {R}_i({\mathbf {x}}_g)$ for $i = 1,\ldots , n^{w}$ are considered. The modal internal force contributions for each snapshot are collected in a snapshot matrix $\underline{\underline{\mathfrak {X}}}_{f} \in \mathbb {R}^{n^\mathrm{g}\times n^{w}\cdot n^\mathrm{s}}$.

32 $$\begin{aligned} \underline{\underline{\mathfrak {X}}}_{f} = \left[ \begin{array}{cccccc} f_1^1({\mathbf {x}}_1) &{} \ldots &{} f_{n^{w}}^1({\mathbf {x}}_1) &{} f_1^2({\mathbf {x}}_1) &{} \ldots &{} f_{n^{w}}^{n^\mathrm{s}}({\mathbf {x}}_1) \\ f_1^1({\mathbf {x}}_2) &{} \ldots &{} f_{n^{w}}^1({\mathbf {x}}_2) &{} f_1^2({\mathbf {x}}_1) &{} \ldots &{} f_{n^{w}}^{n^\mathrm{s}}({\mathbf {x}}_2) \\ \vdots &{} &{} \vdots &{} \vdots &{} &{} \vdots \\ f_1^1({\mathbf {x}}_{n^\mathrm{g}}) &{} \ldots &{} f_{n^{w}}^1({\mathbf {x}}_{n^\mathrm{g}}) &{} f_1^2({\mathbf {x}}_{n^\mathrm{g}}) &{} \ldots &{} f_{n^{w}}^{n^\mathrm{s}}({\mathbf {x}}_{n^\mathrm{g}}) \end{array} \right] \end{aligned}$$

furthermore the resulting integrals are collected in the right-hand side vector $\underline{b} = [ \mathcal {F}^1_1, \ldots , \mathcal {F}^1_{n^{w}}, \mathcal {F}^2_1, \ldots , \mathcal {F}^{n^\mathrm{s}}_{n^{w}} ]^T$ in which $\mathcal {F}^j_i = \int _{\mathcal {V}_\mathrm{m}} f^j_i({\mathbf {x}}) \, \mathrm{d}{\mathcal {V}_\mathrm{m}}$. The integration error for a selected subset of integration points $\mathcal {G} \subset \lbrace 1, 2,\ldots , n^\mathrm{g}\rbrace$ using the associated integration weights $\alpha _i$ for $i \in \mathcal {G}$ is found by

33 $$\begin{aligned} \epsilon _{\mathcal {F}} = \Vert \underline{\underline{J}}_\mathcal {G} \underline{\alpha } - \underline{b} \Vert _2 \end{aligned}$$

where the matrix $\underline{\underline{J}}_\mathcal {G}$ is defined by

34 $$\begin{aligned} \underline{\underline{J}}_{\mathcal {G}} = \left[ \begin{array}{cccc} f_1^1({\mathbf {x}}_{\mathcal {G}_1}) &{} f_1^1({\mathbf {x}}_{\mathcal {G}_2}) &{} \ldots &{} f_1^1({\mathbf {x}}_{\mathcal {G}_{\hat{n}^\mathrm{g}}}) \\ f_2^1({\mathbf {x}}_{\mathcal {G}_1}) &{} f_2^1({\mathbf {x}}_{\mathcal {G}_2}) &{} \ldots &{} f_2^1({\mathbf {x}}_{\mathcal {G}_{\hat{n}^\mathrm{g}}}) \\ \vdots &{} \vdots &{} \ldots &{} \vdots \\ f_{n^{w}}^{n^\mathrm{s}}({\mathbf {x}}_{\mathcal {G}_1})&{} f_{n^{w}}^{n^\mathrm{s}}({\mathbf {x}}_{\mathcal {G}_2}) &{} \ldots &{} f_{n^{w}}^{n^\mathrm{s}}({\mathbf {x}}_{\mathcal {G}_{\hat{n}^\mathrm{g}}}) \end{array} \right] \end{aligned}$$

To find the optimal weights, the following minimization problem has to be solved

35 $$\begin{aligned} \mathcal {G}, \underline{\alpha } = \underset{\mathcal {G} \subset \{1,n^\mathrm{g}\}, \alpha > 0}{\arg \min } \Vert \underline{\underline{J}}_{\mathcal {G}} \underline{\alpha } - \underline{b} \Vert _2 \end{aligned}$$

in which $\hat{n}^\mathrm{g}$ integration points need to be selected and their corresponding (positive) integration weights $\alpha$ need to be determined.

#### Reformulation of the ill-posed minimization problem

Note that problem (35) is again ill-posed for the case in which the integrals of the internal force fields are all equilibrium solutions of the RVE problem. This yields a right-hand side vector $\underline{b} = \underline{0}$, therefore the minimization problem (35) yields the trivial solution to the problem $\underline{\alpha } = \underline{0}$. The ill-posed problem is regularized by integrating an extra function $g({\mathbf {x}}) = 1$ which needs to result in the volume of the RVE $|\mathcal {V}_\mathrm{m}|$ [33].

36 $$\begin{aligned} \underline{\underline{J}}_{\mathcal {G}}&= \left[ \begin{array}{cccc} f_1^1({\mathbf {x}}_{\mathcal {G}_1}) &{} f_1^1({\mathbf {x}}_{\mathcal {G}_2}) &{} \ldots &{} f_1^1({\mathbf {x}}_{\mathcal {G}_{\hat{n}^\mathrm{g}}}) \\ f_2^1({\mathbf {x}}_{\mathcal {G}_1}) &{} f_2^1({\mathbf {x}}_{\mathcal {G}_2}) &{} \ldots &{} f_2^1({\mathbf {x}}_{\mathcal {G}_{\hat{n}^\mathrm{g}}}) \\ \vdots &{} \vdots &{} \ldots &{} \vdots \\ f_{n^{w}}^{n^\mathrm{s}}({\mathbf {x}}_{\mathcal {G}_1})&{} f_{n^{w}}^{n^\mathrm{s}}({\mathbf {x}}_{\mathcal {G}_2}) &{} \ldots &{} f_{n^{w}}^{n^\mathrm{s}}({\mathbf {x}}_{\mathcal {G}_{\hat{n}^\mathrm{g}}}) \\ g({\mathbf {x}}_{\mathcal {G}_1}) &{} g({\mathbf {x}}_{\mathcal {G}_2}) &{} \ldots &{} g({\mathbf {x}}_{\mathcal {G}_{\hat{n}^\mathrm{g}}}) \end{array} \right] \nonumber \\ \underline{b}&= \left( \begin{array}{c} 0 \\ 0 \\ \vdots \\ 0 \\ |\mathcal {V}_\mathrm{m}| \end{array} \right) \end{aligned}$$

#### Cubature weights

It is intractable to evaluate all the $\genfrac(){0.0pt}1{n^\mathrm{g}}{\hat{n}^\mathrm{g}}$ possible combinations. To obtain a good approximation of the minimum a Greedy procedure is applied to select the integration points that are suitable to reduce the integration error. For a given subset of integration points $\mathcal {G}$ the coefficients $\underline{\alpha }$ can be determined using a Least-Square (and when negative values occur a Non-Negative Least-Square) algorithm.

The criterion used to identify candidate points, selects the point that is currently most aligned to the residual of the snapshot integrals. This procedure repeats until enough integration points are selected to drop the residual under the required tolerance for the integration accuracy or the maximum number of gappy integration points $\hat{n}^\mathrm{g}$ is reached.
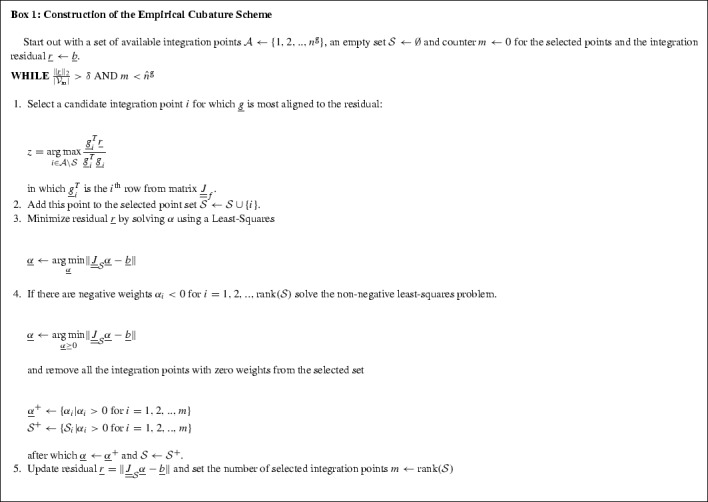


It is not trivial to reconstruct the microscopic stress field from the sampled stresses as the Empirical Cubature Method does not use stress-modes. One can however follow the approach proposed by [33] to reconstruct the microscopic stress field using the weighted reconstruction matrix $\underline{\underline{R}}$ for the selected integration points.

#### Reduced integration of the RVE

The linear momentum balance with reduced internal force (12) is solved by using the Empirical Cubature scheme to resolve the integral. This leads to the following reduced integration scheme

37 $$\begin{aligned} \bar{f}^\mathrm{int}_i(\underline{{W}})&= -\sum _{i = 1}^{\hat{n}^\mathrm{g}} \alpha _i \mathbf {\nabla }^\mathrm{s}\mathbf {R}_i({\mathbf {x}}_{\mathcal {S}_i}) : {\mathsf {\sigma }}_\mathrm{m}({\mathsf {\varepsilon }}_\mathrm{M}, \underline{{W}}, {\mathbf {x}}_{\mathcal {S}_i}, {\mathbf {\xi }}_{\mathcal {S}_i}) \end{aligned}$$

for which the unknown reduced micro-fluctuations $\underline{{W}}$ can be solved using a Newton-Raphson method.

## A comparative analysis of EIM versus ECM

To assess the performance of the hyper-reduced models, a critical comparison is made between the empirical models, the reduced order models and the full-order model of a RVE. Focus is put on the suitability for the hyper-reduced models as a stand-in replacement of the complex full-order model of the RVE in a computational homogenization framework. Small plane-strain is assumed in the model of the RVE. The RVE consists of fibers in a soft matrix. The matrix is modeled using a small-strain elasto-viscoplastic material constitutive law of De Souza Neto et al. [46, p. 148]. The fibers are elastic. The difference in stiffness of the fibers and the matrix is chosen to be a factor 10 to replicate a carbon-fiber epoxy bundle. The properties of both phases in the RVE are listed in Table 1.

**Table 1:** The material properties used for the matrix and fibers of the composite problem. The matrix material is modeled as an elasto-viscoplastic material, whereas the fiber is assumed to be elastic

Material	$E$	$\nu$	$\gamma _0$	$n$	$\sigma ^{\mathrm{y}}_0$	$H$	$m$
	GPa	–	m${}^{-1}$	–	GPa	GPa	–
Matrix	0.1	0.3	0.05	0.05	0.002	0.006	1.2
Fibres	1.0	0.3	–	–	–	–	–

In which $E$ is the Young’s modulus, $\nu$ the Poisson’s ratio, $\gamma _0$ the initial slip rate, $n$ the rate exponent, $\sigma ^{\mathrm{y}}_0$ the initial yield stress, $H$ the hardening parameter and $m$ the hardening exponent. A time-increment $\Delta t= 2.5 \times 10^{-4}$s is used.

**Fig. 3:**
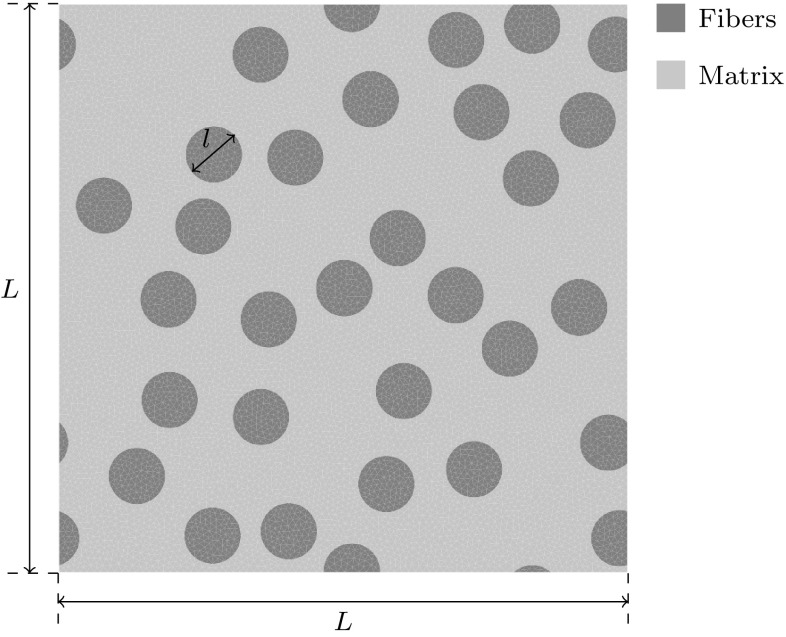
Discretization of the composite microstructure with the fiber and matrix phases. The domain width and height are given by $L = 2.0$. The RVE contains a total of 30 circular fibers with diameter $l = 0.20$. The domain consists of 20,206 triangular elements

The dimensions and topology of the composite microstructure are shown in Fig. 3. The topology is meshed using 20,602 bilinear triangular elements and 10,462 nodes. The full-order problem uses 20,602 integration points for integration and 20,924 degrees of freedom to discretize the microfluctuations.

### Snapshot construction

The reduced order models are initialized using an orthogonal set of macroscopic strains (computed off-line). The full-order model results for the microfluctuations and stresses are stored in the snapshot matrix for each time-increment. The loading paths chosen to initialize the model run from 0 to $0.2 \varepsilon ^\mathrm{eq}_\mathrm{M}$ in 20 steps in the ${\mathsf {\varepsilon }}_{xx}$, ${\mathsf {\varepsilon }}_{yy}$ and ${\mathsf {\varepsilon }}_{xy}$ direction. In this way 60 equilibrium configurations are obtained.

**Fig. 4:**
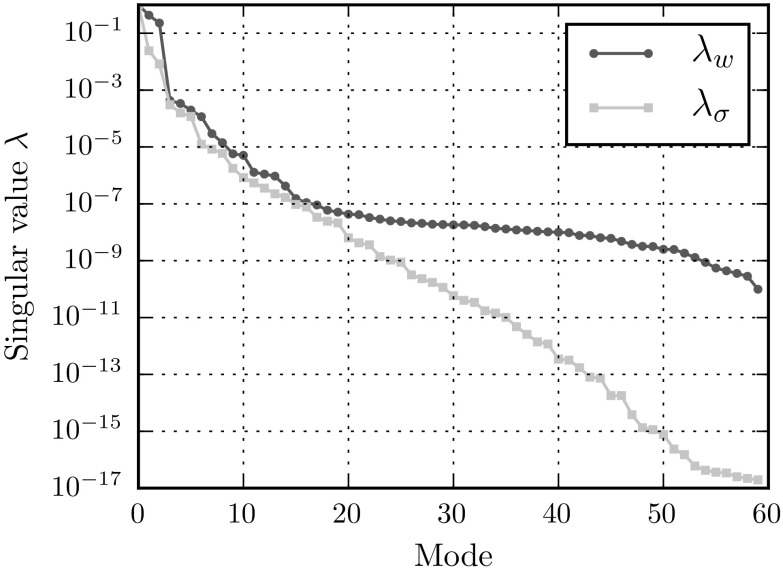
The eigenvalues corresponding to the microfluctuation and stress modes

The microfluctuation and stress-modes are determined from the snapshot matrices using POD. The eigenvalues that correspond to these modes are shown in Fig. 4. To investigate the influence of the number of modes taken into account, two reductions are formulated with a different number of modes. The reduced microfluctuation basis is formed out of the $n^{w}= 10$ and $n^{w}= 20$ most dominant modes of the POD. In the remainder, these reductions will be denoted as the low- and high-fidelity models, respectively. The same number of modes are used to approximate the stress-field in the empirical interpolation model. In correspondence to Hernández [42], the number of sampling points used for accuracy and stability corresponds to the number of stress- and strain-modes respectively.

To generate the empirical cubature scheme, a cut-off tolerance on the integration error is required. The order of magnitude of the interpolation error is predicted by the sum of the squared eigenvalues of the truncated modes of the stress basis. These interpolation errors $\delta _1 = 10^{-3}$ and $\delta _2 = 10^{-4}$ are used as tolerances to cut of the integration point selection algorithm.

In the remainder of this paper, three different techniques will be compared: (1) standard reduced order (with full integration), (2) empirical interpolation and (3) empirical cubature models. A summary of all the specifications of the (Hyper-)Reduced Order Models in terms of modes and sampling points are given in Table 2.

**Table 2:** The number of microfluctuation modes used in the reduced and hyper-reduced models, the number of modes used for the empirical interpolation of the stress-field and the number of sample points in the empirical cubature model required to achieve the specified integration tolerance

	ROM	EIM	ECM
	$n^{w}$	$n^{\sigma }$	$\delta \rightarrow \hat{n}^\mathrm{g}$
Low-fidelity	10	10	$10^{-3} \rightarrow$ 67
High-fidelity	20	20	$10^{-4} \rightarrow$ 321

In order to assess the achieved approximative qualities of the reduced models, the microfluctuations and micro- and macroscopic stresses are compared to the full-order model. In the reduced order model there is no loss of information present during the integration of the stress-field to obtain the internal forces. Therefore, this model can be regarded as the best-approximation for the applied reduced basis and serves as a reference model to investigate the performance of the hyper-reduced models. The achieved time reduction will be evaluated by comparing the CPU-time required to perform the computations to the CPU-time required to solve the original full-order model.

### Uniaxial loading

The accuracy of the empirical methods are first investigated for one of the load-cases used to generate the snapshots (${\mathsf {\varepsilon }}_\mathrm{M}^{xx}$). This case serves to highlight the influence of the reduced stress integration in the (hyper-)reduced order models. Note that the modes required to represent the microfluctuation and the stress-fields are then present up to the cut-off tolerance of the POD basis. The effects of the stress-field reconstruction for EIM and integral reconstruction for ECM using gappy sampling on the final deformation and stress fields will therefore be dominant.

**Fig. 5:**
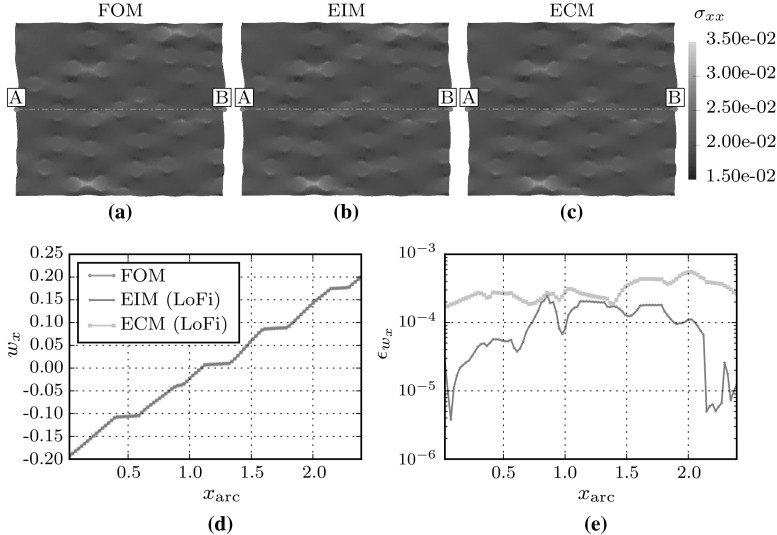
The deformed RVE under macroscopic strain ${\mathsf {\varepsilon }}_{\mathrm{M},xx} = 0.2$ resolved with the full-order method (**a**), EIM (**b**) and ECM (**c**). The microfluctuations along line A–B and the relative error $\epsilon _{w} = \frac{\bar{w}_x - w^\mathrm{FOM}_x}{w^\mathrm{FOM}_x}$ plotted in (**d**) and (**e**) respectively

The Representative Volume Element is loaded with a macroscopic strain ${\mathsf {\varepsilon }}^{\mathrm{M}}_{xx}$ from 0 to 0.2 in 20 time-increments (each with the specified time-step $\Delta t$). Figure 5 shows the RVEs loaded up to a macroscopic strain of ${ \varepsilon } _{\mathrm{M},xx} = 0.2$. The deformed RVEs solved by the full-order model and the low-fidelity hyper-reduced models are plotted in Fig. 5a–c. Not surprisingly, the essential deformation modes for this case are all captured by the low-fidelity models. The microfluctuation $w_x$ along the line A–B and the errors, defined as

$$\begin{aligned} \epsilon _{w} = \frac{\bar{w}_x(x_\mathrm{arc}) - w_x(x_\mathrm{arc})}{w_x(x_\mathrm{arc})} \end{aligned}$$

for the hyper-reduced models, are plotted in Fig. 5d, e respectively. The error of the hyper-reduced RVEs are both of order $\mathcal {O}\left( {10^{-4}}\right)$ or lower.

**Fig. 6:**
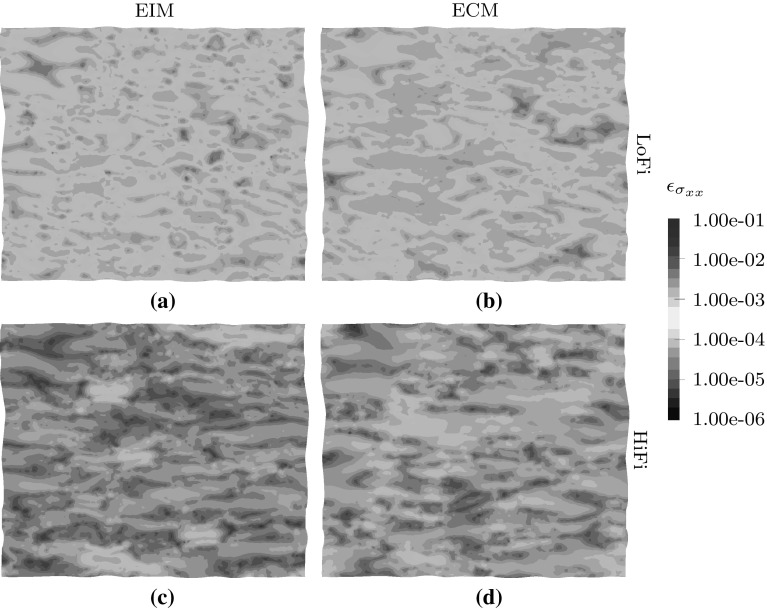
Residual fields of the microscopic stress component ${\mathsf {\sigma }}_\mathrm{xx}$ in the loading direction $\mathrm{xx}$ for the hyper-reduced models w.r.t. the full-order solution. The first row shows the residual for the Lo-Fidelity EIM (**a**) and ECM (**b**) models and the second row shows the residual for the Hi-Fidelity EIM (**c**) and ECM (**d**) models

Secondly, the errors in the microscopic stress field are analyzed. The stress fields $\bar{{\mathsf {\sigma }}}({\mathbf {x}})$ resulting from the (hyper-)reduced models are compared to the stress field ${\mathsf {\sigma }}({\mathbf {x}})$ of the FOM model. It should be noted that the local-stress field for the ECM method is obtained by creating the reconstruction operator $\underline{\underline{R}}$ for the sampled points. The error in the stress in the xx-direction $\epsilon _{ { \sigma } _\mathrm{xx}}$ is defined as

38 $$\begin{aligned} \epsilon _{ { \sigma } _\mathrm{xx}} = \frac{\int _{\mathcal {V}_\mathrm{m}} \Vert \bar{ { \sigma } }_\mathrm{xx}({\mathbf {x}}) - { \sigma } _\mathrm{xx}({\mathbf {x}}) \Vert _2 \, \mathrm{d}{\mathcal {V}_\mathrm{m}}}{\int _{\mathcal {V}_\mathrm{m}} \Vert { \sigma } _\mathrm{xx}({\mathbf {x}}) \Vert _2 \, \mathrm{d}{\mathcal {V}_\mathrm{m}}} \end{aligned}$$

The stress residuals are depicted in Fig. 6. For both the LoFi and the HiFi model, the EIM method performs better than the ECM method. This difference results from the extra information that the empirical interpolation model uses from the stress-modes. Since these modes contain a lot more spatial details, the stress-field is captured more accurately.

Furthermore, it can be concluded that the error bounds, although they are lower, do not differ significantly between the LoFi and the HiFi models. The volume averaged error in the stress dropped significantly for both models.

**Fig. 7:**
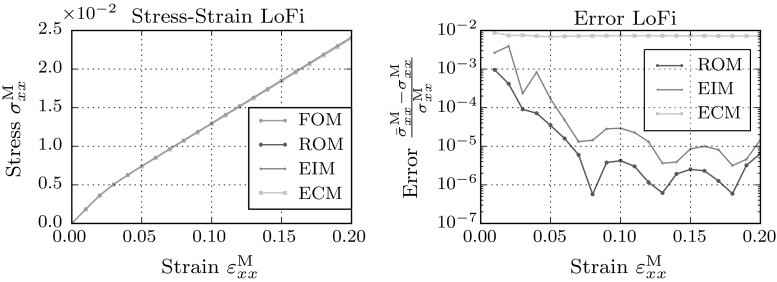
Macroscopic stress–strain plot in xx-direction for the full-order model and the low-fidelity reduced order models

**Fig. 8:**
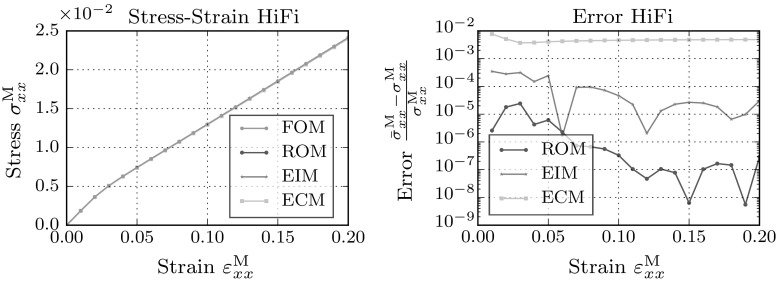
Macroscopic stress–strain plot in xx-direction for the full-order model and the high-fidelity reduced order models

The macroscopic stress resulting from full- and (hyper-)reduced order models are plotted in Figs. 7 and 8. For this load-case the resulting macroscopic stresses show no large deviations from the full-order result. Interestingly, the errors plotted on the right hand side, show a clear difference in the EIM and ECM approximation of the macroscopic stress. The EIM approximation follows the error trend of the reduced order model, while the error made by the ECM model is several decades higher than the error of the reduced order model.

**Fig. 9:**
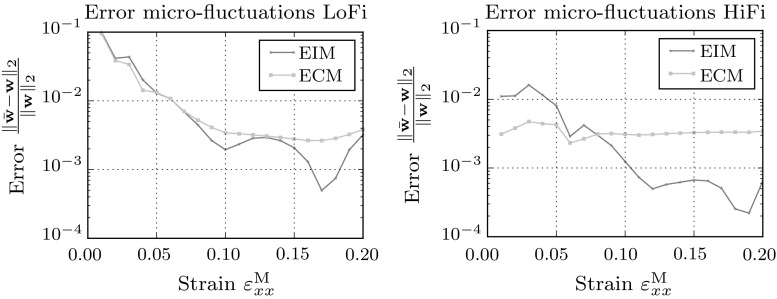
Error in the integrated micro-fluctuation fields for the full-order model and the hyper-reduced models

The error in the microfluctuations of the hyper-reduced models ($\bar{{\mathbf {w}}}$) relative to the full-order model (${\mathbf {w}}$) is plotted in Fig. 9. The error is defined as follows:

39 $$\begin{aligned} \epsilon _{ { {w} } } = \frac{\int _{\mathcal {V}_\mathrm{m}} \Vert \bar{{\mathbf {w}}}({\mathbf {x}}) - {\mathbf {w}}({\mathbf {x}}) \Vert _2 \, \mathrm{d}{\mathcal {V}_\mathrm{m}}}{\int _{\mathcal {V}_\mathrm{m}} \Vert {\mathbf {w}}({\mathbf {x}}) \Vert _2 \, \mathrm{d}{\mathcal {V}_\mathrm{m}}} \end{aligned}$$

The error in the microfluctuation fields decreases with increasing number of strain modes. The reduction in error is however not equal for each increment. The error in the empirical cubature method between ${\mathsf {\varepsilon }}^\mathrm{M}_{xx} = 0.00$ and 0.10 is significantly reduced (where most of the non-linearity is concentrated) by the added modes and integration points.

It is noteworthy that the reduction by the empirical interpolation method seems to outperform the empirical cubature method only to small extent, which is remarkable since the difference in approximation errors of the macroscopic stress for both models differed by several decades.

The computation times for all models are assessed on a single CPU (Intel^®^ Xeon^®^ CPU E5-2667 v3 processor @ 3.20 GHz), without parallelization to keep the comparison clear. The speed-up with respect to the full-order model are presented in Table 3. The speed-up for the ROM model is relatively insensitive to the number of modes used and the gain in computational time is small compared to the full-order model of the RVE. This illustrates the poor performance of classical ROM for non-linear reduced models. The EIM model speeds up between a factor 112 and 123 times. The speed-up in the ECM models are between 73 and 113 times. The ECM model is somewhat slower due to the larger amount of integration points required.

**Table 3:** Speed-up of the tensile simulations running on a single CPU

Case	Method	Speed-up	
		LoFi	HiFi
Tensile	FOM	$1\times$	$1\times$
	ROM	$1.32\times$	$1.12\times$
	EIM	$111.92\times$	$123.65\times$
	ECM	$113.33\times$	$73.60\times$

**Fig. 10:**
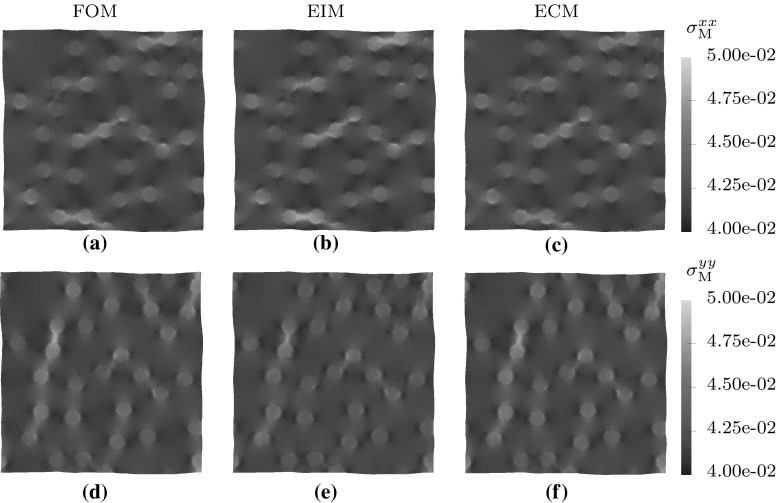
The resulting stresses in *xx* and *yy* direction of the full- and reduced-order model of the RVE under biaxial loading

**Fig. 11:**
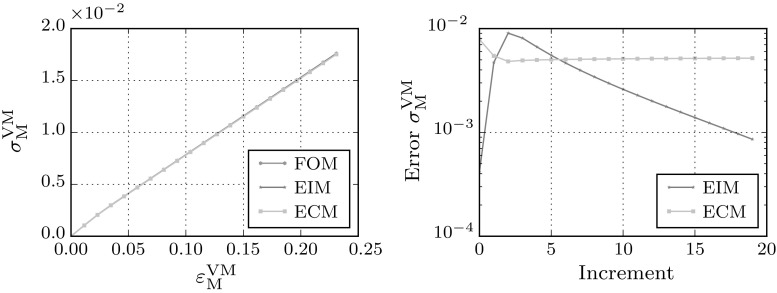
Macroscopic stress–strain curve for biaxial loading in *xx* and *yy* direction for strains ranging from ${\mathsf {\varepsilon }}_\mathrm{M}= 0.0$ to 0.2 (left) and the relative error in the von Mises stress $\sigma ^\mathrm{VM}_\mathrm{M}$ of both hyper-reduced models with respect to the full-order model (right)

### RVE under biaxial loading

A biaxial load is applied next to test the performance of the reduced models under loads not present in the snapshots used to train them. The macroscopic strain is increased to $${\mathsf {\varepsilon }}_\mathrm{M}=\left[ \begin{array}{ll} 0.2, &{} 0.0 \\ 0.0, &{} 0.2 \end{array}\right] $$ in 20 equal load-increments.

From the FOM and ECM stress fields presented in Fig. 10, it is clear that all the dominant plastic zones are recovered by the reduced model. The stress–strain curves of the biaxial load-case presented in Fig. 11, indicate that in the linear regime the EIM model outperforms the ECM model. Since the corresponding modes are present, the stress approximation of the interpolating model is more accurate. However, when entering the plastic regime, the ECM model seems to perform at consistent error level, while the error in the macroscopic von-Mises stress predicted by the EIM model increases due to lack of the sampling points required to accurately interpolate the stress modes for this load case.

**Table 4:** Speed-ups of the biaxial simulations on a single CPU

Case	Method	Speed-up
		HiFi
Biaxial	FOM	$1\times$
	ROM	$1.04\times$
	EIM	$88.42\times$
	ECM	$53.57\times$

**Fig. 12:**
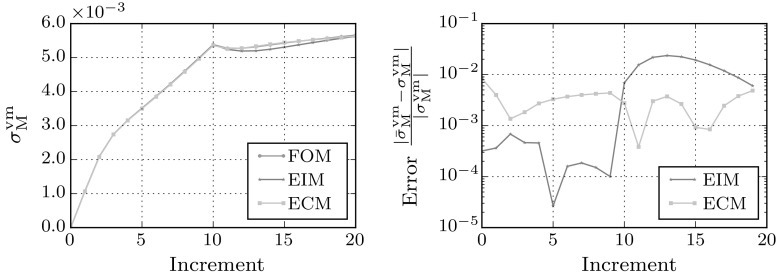
Macroscopic stress–strain curve for sequential path dependent loading in two directions: first 10 strain increments up to ${\mathsf {\varepsilon }}^\mathrm{M}_\mathrm{xx} = 0.1$ followed by 10 strain increments up to ${\mathsf {\varepsilon }}^\mathrm{M}_\mathrm{xy} = 0.1$

The cpu-times of the simulations are given in Table 4. The hyper-reduced methods EIM and ECM are 54 and 88 times faster than the full-order method respectively. The difference in speed-up between the empirical interpolation and the empirical cubature method can be explained by the extra gappy points required by ECM to obtain accurate solutions.

### Path dependency

To investigate the sensitivity of both reduced models to history dependent behavior, the models are preloaded with 10 increments of strain in the xx-direction $${\mathsf {\varepsilon }}^\mathrm{M}= \left[ \begin{array}{ll} 0.1,&{} 0.0 \\ 0.0, &{} 0.0 \end{array}\right] $$. After preloading the RVE, the model is loaded under shear $${\mathsf {\varepsilon }}^\mathrm{M}= \left[ \begin{array}{ll} 0.0,&{} 0.5 \\ 0.5, &{} 0.0 \end{array} \right] $$. The macroscopic von Mises stress at each increment is plotted in Fig. 12.

In the first regime, the EIM model is more accurate than the ECM model due to availability of the correct modes. When entering the shear strain regime at increment 10, the ECM method starts to perform better than the EIM method. The EIM model depends strongly on the stress-modes found during the off-line phase. Since the reduced-model was only trained using RVEs with virgin material without prior loading history, the required modes are not present to accurately capture the path-dependent stress fields. The small dependence on stress modes makes the ECM easier to train. Obviously, strain modes are also different for different loading paths, whereas the modes found in the snapshots include monotonic paths only. For this small-strain model, this mainly effects the stresses, since the ECM does not show a significant drop in accuracy after increment 10.

**Table 5:** Speed-ups of the path dependent simulations on a single CPU

Case	Method	Speed-up
		HiFi
Path dependent	FOM	$1\times$
	ROM	$1.25\times$
	EIM	$131.42\times$
	ECM	$69.50\times$

The speed-up achieved by the (hyper-)reduced models for the path-dependent case is presented in Table 5. The speed-ups achieved during the path-dependent loading are comparable to the speed-up factors found in the biaxial test-case presented in Table 4.

### Cyclic loading

Finally, the behavior of the models under repetitive loading is analyzed. The RVE is exposed to a cyclic load in the *xx*-direction, shifting its history increasingly further away from its virgin state. The loading direction corresponds to the snapshots, excluding the influence of any interpolation in between different loading directions.

**Fig. 13:**
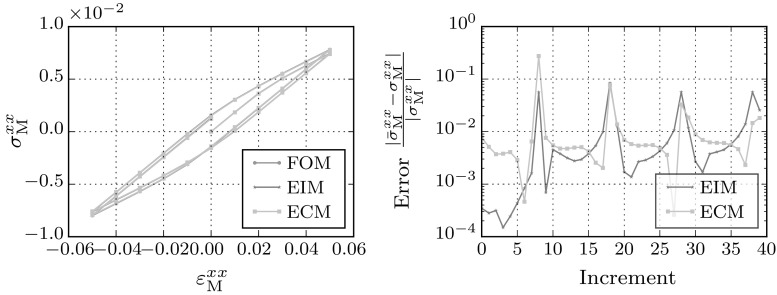
Macroscopic stress–strain curve for cyclic loading in *xx* direction between strains ${\mathsf {\varepsilon }}^\mathrm{M}_\mathrm{xx} = -0.1$ and 0.1

The stress–strain curve is shown in Fig. 13. The error in between the full-order model and the hyper-reduced models increases slowly over the increments. Due to the single loading direction the difference between the EIM and the ECM method is much less pronounced than in the path dependent case. When switching between tensile and compressive loading, the error shows significant jumps indicating that the unloading stage, which is absent in the off-line initialization stage, is hard to capture for both the ECM and EIM models.

**Table 6:** Speed-ups of the cyclic loading simulations on a single CPU

Case	Method	Speed-up
		HiFi
Cyclic	FOM	$1\times$
	ROM	$1.19\times$
	EIM	$114.95\times$
	ECM	$56.84\times$

The speed-up achieved by the (hyper-)reduced models for the cyclic load-case are presented in Table 6. Although the runtime of the simulations is longer due to the extra increments, the speed-ups achieved during the path-dependent loading are comparable to the speed-up factors found in the previous test-cases presented in Tables 4 and 5.

## Conclusions

Both the empirical interpolation method and empirical cubature method yield a significant reduction in computation time and memory footprint compared to traditional ROM. Both models are capable of interpolating between the sampled deformations as shown for the biaxial load-case. When the load-case includes unsampled states of the history parameters, the empirical interpolation method result in errors above 1% for the macroscopic stress with respect to the full-order model. The empirical cubature method is less sensitive to the extrapolation of the snapshot space, since it only depends on the strain modes and not on the stress-modes.

The main asset of the ECM method is that it naturally preserves the stability of the full-order model and therefore does not require the added stabilization needed for the EIM method. However, with the stabilizing integration points added, the EIM models showed good convergence for all the cases demonstrated in Sect. 5, using the (heuristic) criterion of $n^{w}$ stabilization points suggested in literature.

The empirical cubature method requires more computational time and has a larger memory footprint than the empirical interpolation method due to the extra integration points needed to accurately capture the behavior of the full-order model. The EIM method outperforms the ECM method when on-line computational efficiency is considered due to the extra information it has available in the stress-modes. However, this dependence on stress-modes makes EIM also more vulnerable for ill-sampled path dependent problems, as demonstrated in the path-dependent and cyclic examples.

The largest reduction in required memory and computation time can therefore be achieved using the empirical interpolation method in a computational homogenization setting, due to the smaller amount of memory required to store the history of the sampling. To get accurate results, the high-dimensional snapshot space needs to be sampled sufficiently well such that the paths found in the macroscopic problem can be interpolated sufficiently accurate by the EIM model.

Herein lies an open challenge, since the material model used in the above examples requires 7 history parameters and 3 strain parameters to calculate the macroscopic stress, the sample space of the model is 10 dimensional. It is therefore not trivial to obtain a sufficiently dense sampling of the parameter-space to capture the path-dependent macroscopic stress.

In the considered computational homogenization problems, the microscopic RVE has to be evaluated numerous times in the deformation and history space. It is therefore necessary to construct an accurate yet compact snapshot space. The empirical interpolation method is expected to be sufficiently accurate for many engineering purposes (within 10% error). However, when a higher accuracy is required, ECM methods are more promising since they are less prone to produce large interpolation errors. In problems with a strong path-dependence, it is more straightforward to construct an accurate snapshot space for ECM models.

## Footnotes

1 Note that the singular values $\sigma _i$ resulting from *singular value decomposition* (SVD) are related to the eigenvalues of the proper orthogonal decomposition via $\sigma _i = \sqrt{\lambda _i}$

2 This relation holds only for fluctuation fields that are present in the snapshot matrix.
